# Criteria for prioritization of HIV programs in Viet Nam: a discrete choice experiment

**DOI:** 10.1186/s12913-017-2679-0

**Published:** 2017-11-13

**Authors:** Ali Safarnejad, Milena Pavlova, Vo Hai Son, Huynh Lan Phuong, Wim Groot

**Affiliations:** 1 Maastricht Graduate School of Governance, Maastricht University, P.O. Box 616, 6200 MD Maastricht, Netherlands; 2 Department of Health Services Research; CAPHRI, Maastricht University Medical Center, Faculty of Health, Medicine and Life Sciences, Maastricht University, Maastricht, Netherlands; 3 Viet Nam Authority of HIV/AIDS Control (VAAC), Ministry of Health, Hanoi, Vietnam; 4 The Joint United Nations Programme on HIV/AIDS (UNAIDS), Hanoi, Vietnam

**Keywords:** HIV, AIDS, Vietnam, DCE, Priority setting

## Abstract

**Background:**

With the decline in funding for Viet Nam’s response to the HIV epidemic, there is a need for evidence on the criteria to guide the prioritization of HIV programs. There is a gap in the research on the relative importance of multiple criteria for prioritizing a package of interventions. This study elicits preferences and the trade-offs made between different HIV programs by relevant stakeholders and decision-makers in Viet Nam. It also pays attention to how differences in social and professional characteristics of stakeholders and their agency affiliations shape preferences for HIV program criteria in Viet Nam.

**Methods:**

This study uses self-explicated ranking and discrete choice experiments to determine the relative importance of five criteria - effectiveness, feasibility, cost-effectiveness, rate of investment and prevention/treatment investment ratio - to stakeholders when they evaluate and select hypothetical HIV programs. The study includes 69 participants from government, civil society, and international development partners.

**Results:**

Results of the discrete choice experiment show that overall the feasibility criterion is ranked highest in importance to the participants when choosing a hypothetical HIV program, followed by sustainability, treatment to prevention spending ratio, and effectiveness. The participant’s work in management, programming, or decision-making has a significant effect on the importance of some criteria to the participant. In the self-explicated ranking effectiveness is the most important criterion and the cost-effectiveness criterion ranks low in importance across all groups.

**Conclusions:**

This study has shown that the preferred HIV program in Viet Nam is feasible, front-loaded for sustainability, has a higher proportion of investment on prevention, saves more lives and prevents more infections. Similarities in government and civil society rankings of criteria can create common grounds for future policy dialogues between stakeholders. Innovative models of planning should be utilized to allow inputs of informed stakeholders at relevant stages of the HIV program planning process.

**Electronic supplementary material:**

The online version of this article (10.1186/s12913-017-2679-0) contains supplementary material, which is available to authorized users.

## Background

The first case of HIV infection in Viet Nam was reported in December 1990 in Ho Chi Minh City. By December 2003, 76,180 infections were reported in Viet Nam and 6550 people had died of AIDS related causes [1, 2]. By December 2015, there were 255,000 people living with HIV and over 128,000 people had died from AIDS-related illnesses since the start of the epidemic [3].

Viet Nam’s HIV epidemic is concentrated among three key population groups defined by risk behaviors and a high prevalence of HIV: people who inject drugs, men who have sex with men and female sex workers [4]. The main route of transmission is through injecting drugs followed by sexual transmission. By 2015, the estimated number of new infections had decreased by 50% from the peak of the epidemic in 2003, thanks to prevention initiatives for key populations, including the provision of clean needles and syringes, provision of condoms, methadone maintenance therapy, and antiretroviral treatment [3, 5].

International donors have provided substantial support to Viet Nam’s HIV response. In 2005, The U.S. President’s Emergency Plan for AIDS Relief (PEPFAR), Asian Development Bank, World Bank, UK Department for International Development (DFID), The Global Fund to fight AIDS, Tuberculosis and Malaria (GFATM), and Australian Agency for International Development (AusAID) were financing a significant portion of the HIV programs [6]. Viet Nam’s recent reclassification as a lower middle-income country, introduced a challenge in financing of the HIV response [7] because most donors provide more official development assistance to lower income countries than other income groups [8]. By 2015, only GFATM and PEPFAR remained in Viet Nam to provide funding for the HIV response, including funding 95% of the costs of Anti-retroviral Treatments [9]. The national HIV program faced sustainability issues due to the substantial decline in external donor funding commitments beyond 2017 [10]. The rapid phase out of donors has alarmed the government of Viet Nam. Therefore, the Deputy Prime Minister has called on the international donor community to give the country more time to transition to domestic funding of the HIV response, including use of social health insurance for curative care [11].

Global shortfalls in funding for the HIV response make it unlikely that the withdrawal of international funding will slow down in Viet Nam. The 2016 report of the Kaiser Family Foundation (KFF) and UNAIDS indicated that donor funding to support the HIV response efforts in low- and middle-income countries, had declined for the first time in five years [12]. “Donors faced many competing funding demands, including humanitarian emergencies and the refugee crisis, all against a backdrop of fiscal austerity in a number of countries”, explained Jen Kates, KFF Vice President and Director of Global Health and HIV Policy [13].

In 2012, after a decade of financial support to Viet Nam’s HIV response, the World Bank and DFID ended their funding. Their recommendation for Viet Nam before exiting was to refocus the government funding of HIV prevention programs on provinces in the country based on epidemiological impact, infrastructure, and ability of communities to mobilize resources [14]. In 2014, Viet Nam’s Ministry of Health, with support from UNAIDS, developed the Investment Case, which identified priorities and the most effective approaches for the National HIV response [5]. Although commendable, the Investment Case limited the prioritization criteria to effectiveness and cost-effectiveness. And while other criteria, like sustainability, were mentioned as principles, there was no explicit use of other criteria for priority setting. Faced with shrinking donor funds, future prioritization initiatives in Viet Nam may lean further towards prioritizing the cost-effectiveness of the HIV program package, while neglecting other relevant criteria.

Given the limited funding, there is a growing interest in generating evidence on the criteria to guide priority setting in the HIV response [15–20]. A number of studies have considered multiple criteria explicitly to prioritize specific prevention interventions [18, 21, 22] or HIV treatment [23, 24]. In Indonesia and Pakistan, a broad set of HIV interventions in the national HIV response were considered, and stakeholders were involved in self-explicating the importance of criteria for priority setting [25, 26]. A Thai study used more rigorous experimental methods to rate criteria that guide priority setting, involving decision-makers as well as stakeholders living with or at higher risk of HIV, thereby reducing the bias in self-reported importance of criteria [15]. The design of the Thai study considered prioritization of targeted interventions rather than the program package of interventions. In a number of the mentioned studies, diverse stakeholder groups were convened to consider one criterion for prioritizing HIV interventions, while other studies convened a limited group to consider multiple criteria. The aforementioned studies were limited in terms of a narrow focus on a limited set of interventions, a lack of involvement of key stakeholders, or use of less rigorous study designs. These limitations collectively have left a gap in the research on the relative importance of multiple criteria for prioritizing a package of interventions.

This study elicits preferences and the trade-offs made between different HIV programs by relevant stakeholders and decision-makers in Viet Nam. In other words, given several criteria for deciding on a HIV program, how much of one criterion are they willing to give up for improvements in another criterion. We also pay attention to how differences in social and professional characteristics of stakeholders and their agency affiliations shape preferences for HIV program criteria in Viet Nam. This study is innovative in its use of discrete choice experiments (DCE) and self-explicated ranking to establish the relative importance of a set of criteria for prioritizing Viet Nam’s HIV response.

DCE are based on well-tested theories that provide an explanation of choice behavior [27]. These experiments place individuals in scenarios where they have to make a choice between options presented to them. In that decision-making process, the individual considers the criteria that define the options, and the trade-off in criteria they are going to make in choosing one option over another option. Data from the individual choices can then be used to quantify the relative importance of the criteria. DCE have been used extensively to examine preferences and priorities in health care (e.g., [28–32]).

This study contributes to Viet Nam’s HIV policy-making by clarifying what criteria are being considered in prioritizing the programs in the National HIV response, and how different stakeholders view the importance of those criteria. Other countries can adopt this transparent and accountable process during their national HIV planning process and in prioritizing their HIV funding proposals to donors.

## Method

This study used two methods to elicit stakeholders’ preferences for and choices of HIV programs. The first method was a straightforward self-explicated ranking of criteria. The second method used the DCE method to determine the relative importance of criteria to stakeholders when they evaluate and select HIV programs.

DCE is a type of hypothetical experiment that is widely used in the health field to quantify preferences. The experiment imitates a situation when a stakeholder must make a choice between two or more options. Each option has the same set of attributes as the other option but the values of these attributes are varied to make the option different from the other. In this study, the DCE attributes are referred to as “criteria”, which is the common term used in priority setting and operations research. The variance of the criteria was fixed to two levels. To reduce the cognitive burden required by the respondents (stakeholders and decision-makers), the number of choice scenarios presented to them was minimized using orthogonal arrays. Each choice scenario contained two HIV programs. During the experiment, the choice scenarios were presented to the respondents consecutively, and the respondents were asked to take their time to select the HIV program they preferred most in each choice scenario.

### Selection of criteria and levels

The most frequently used criteria identified in a systematic review of literature served as a basis for the DCE in this study [33]. The full set of 18 criteria in the systematic review were reviewed with HIV experts working in Viet Nam to assess their relevance to the country’s HIV epidemic and response. Based on that assessment, five criteria of a HIV program were selected: effectiveness, feasibility, cost-effectiveness, rate of investment and prevention/treatment investment ratio. The first three of these criteria were characterized as program outcomes. The last two criteria were characterized as program inputs.

All criteria had dichotomous levels. These criteria levels were generated based on investment case scenarios developed for Viet Nam in 2014 [5]. The investment case scenarios were developed using the AIDS Epidemic Model (AEM). AEM is a standard estimation and projection tool used in modeling the epidemic in countries with concentrated epidemics. The AEM uses input program and epidemiological data to estimate the future impact of proposed policies and program coverage levels, as well as the size of the investment required. One of the assumptions that was used in the selection of criteria levels, was that the AEM projections went until 2030, when international and national goals are to be met. Additionally, it was assumed in the AEM estimations that funding would be capped to the current level of spending, and should not be expected to increase beyond current levels. Table 1 presents all criteria, their levels and coding for the analysis.

**Table 1:** Criteria, criteria levels and their coding

Criteria & Levels	Basic Profile	Orthogonal array							Criteria difference						
		A	B	C	D	E	F	G	ΔA	ΔB	ΔC	ΔD	ΔE	ΔF	ΔG
Effectiveness	0	1	0	0	0	1	1	1	1	0	0	0	1	1	1
1 = five million years of life saved															
0 = four million years of life saved															
Cost-effectiveness	1	1	0	0	1	0	1	0	0	−1	−1	0	-1	0	-1
0 = $5 return for every $1 invested															
1 = $6 return for every $1 invested															
Sustainability	1	0	1	0	0	1	1	0	-1	0	-1	-1	0	0	-1
0 = spending constantly increases															
1 = spending increase then decrease															
Treatment/Prevention	1	1	1	0	0	0	0	1	0	0	-1	-1	-1	-1	0
0 = greater investment in treatment															
1 = greater investment in prevention															
Feasibility	0	0	1	0	1	0	1	1	0	1	0	1	0	1	1
0 = low feasibility															
1 = high feasibility															

### Study instrument, DCE design and DCE validity

Prior to the DCE, the questionnaire presented an operational definition of each criterion, and asked respondents to manually rank the criteria according to the importance they attached to each of the criteria. This step allowed the comparison of self-explicated ranking with the DCE weighted ordering of the criteria. It also allowed a common understanding of the criteria by the participants prior to starting the DCE.

To limit the number of combinations of scenarios and avoid information overload by participants in the DCE, criteria were limited to five with two levels each (see Table 1), which resulted in a total of 32 (2^5^) possible profiles. A subset of those 32 profiles was chosen on the basis of a fractional experimental designs library of orthogonal arrays [34]. The fractional factorial design included a subset of eight profiles and strength of two that allowed the estimation of all main effects. The construction of eight profiles and related coding is presented in Table 1.

One of the eight profiles defined by the fractional factorial design was selected as the basic profile, and the rest were used as alternative profiles. The basic profile was selected to create realistic and challenging decision situations for participants in the experiment. Then seven choice scenarios were created where respondents were asked to choose between the basic profile and one of the alternative profiles based on their preference for the criteria for prioritizing the HIV response. This DCE design minimized the number of comparisons (choice scenarios) respondents had to make while giving a reasonable estimation of the main effects.

The survey that presented the DCE also collected information on the respondent’s social and professional characteristics. These data were used to assess how individual factors influence decision-maker’s stated preferences. The assessed characteristics included age, sex, nationality, professional experience, and engagement in decision-making in Viet Nam’s HIV response. The complete survey questionnaire in English and Vietnamese are provided in Additional files 1 and 2 respectively.

### Study setting and participants

The DCE was conducted in Viet Nam, where the HIV response involves development partners, civil society, and the central and provincial state institutions. The study aimed to reach 60 participants who had been involved, or currently are involved, in the decision-making in Viet Nam’s HIV response, with similar number of respondents from government, international development partners, and civil society organizations respectively. The motivation behind selecting these three groups was that they encompass the actors who have a stake in the decision-making in the HIV response. These three groups are referenced by the Greater Involvement of People Living with HIV (GIPA) principle formalized at the 1994 Paris AIDS Summit [35], and are also reflected in the membership structure of the Country Coordinating Mechanism of the GFATM [36]. The proportion of individuals in the three groups may not represent the same proportion of individuals who were involved in decision-making processes in Viet Nam at the time of the study, however. For example, the GFATM Country Coordinating Mechanism involves 7 individuals from government, 6 from international development partners, and 11 from civil society organizations [37]. The steering committee for development of the investment case scenarios involved 3 individuals from the government, 6 individuals from international development partners, and no civil society members, although the latter were involved in consultations to obtain inputs “on their priorities for the response in future” [5]. The National Strategic Plan for HIV/AIDS Prevention and Control in Viet Nam was developed with active participation of the three groups, but the extent of that participation or process of prioritization has not been documented [1, 38].

Participants were identified in a two-stage selective sampling of individuals knowledgeable or responsible for decision-making. In the first stage, the researchers identified seed individuals in government, civil society, and development partners. Seed individuals are initial study participants who recruit their social or professional peers [39]. In the second stage, the seed individuals initially nominated twenty individuals each within their network to potentially respond to the questionnaire. If some invited individuals did not respond or were unable to respond to the questionnaire, the seed individuals sent five new invitations to potential respondents. This process continued until the quota of 60 participants with similar number of respondents from the three groups was reached. The nominated individuals received a web link to anonymously respond to the survey. All eligible participants were informed of the purpose of the study and notified that they can exit the survey at any time and choose to have all their answers be deleted. Thus, informed consent to participate was provided by each respondent.

### Pilot of the study

The DCE was piloted with four participants. As a result of the pilot, the ordering of choice scenarios was revised to start with the simplest task (with two differences between the two profiles) to the most difficult task (with four differences between the two profiles). The choice scenarios were also formatted to be displayed horizontally, compared to the vertical presentation customary in DCE. This allowed the respondents to give equal attention to all criteria of the profile, and reduced the dominance of the criterion on top. Finally, where graphs were used to illustrate the meaning of criteria, footnotes were added to further clarify those graphs.

### Survey administration, data collection and analysis procedure

The DCE survey was administered electronically on the LimeSurvey platform using a standardized questionnaire. The participant choices were coded 0 if the basic profile was selected as the preferred profile and coded 1 if the alternative profile was selected as the preferred profile.

The data collected were inserted in Excel and cleaned of any inconsistent answers to the DCE scenarios. Two respondents provided a combination of responses that were categorized as inconsistent answers. Firstly, if a respondent selected a high feasibility program over a cost-effective program (choice scenario 2), then selected a program with constantly increasing costs and high proportion of spending on treatment over a cost-effective program (choice scenario 3), and then selected a low feasibility, low proportion of spending on treatment, and decreasing cost program which is highly cost-effective (choice scenario 4), that respondent’s answers were deemed inconsistent. Secondly, if a respondent selected an effective program over a program with decreasing costs (choice scenario 1), then selected a highly feasible program over a cost-effective program (choice scenario 2), and then selected a cost-effective and decreasing cost program over a feasible and effective program (choice scenario 7), that respondent’s answer were deemed inconsistent. The DCE results of the two respondents who provided inconsistent responses were removed from the data set.

The cleaned dataset was imported into Stata for analysis. For the analysis of the DCE data, a binary logit regression with random intercepts was used. First, the main-effect model was estimated using the responses of all respondents. Then the responses were disaggregated by group membership – government, civil society, development partners – and the main effects model was estimated for each group, and the results were compared. Data from the self-explicated ranked criteria were analyzed using descriptive statistics, and ordered by the modes of the criteria ranks. The DCE results were compared to the self-explicated ordering of criteria in qualitative terms.

Social and professional characteristics may have an effect on the choices of stakeholders and decision-makers. Descriptive statistics were calculated for social and professional characteristics of the respondents. After estimating the main effects, interactions of the criteria with the social and professional characteristics of the respondents were added to the model. A backward stepwise regression procedure was conducted to obtain a reduced model consisting of statistically significant independent variables (*p* < .05).

## Results

Of the 82 people invited to participate in the survey, 69 (84%) agreed and completed the survey. Survey respondents include 31 (44%) females and 38 (56%) males. The majority of respondents (44%) are between 41 and 50 years old. The majority of respondents (76%) are Vietnamese nationals. There are nearly equal proportions of respondents from civil society, government, and development partners, with 20 (29%), 26 (38%), and 23 (33%) representatives respectively. Most respondents (91%) are involved in decision-making and more than one half (65%) are responsible for decision-making. Information on all social and professional characteristics is provided in Table 2.

**Table 2:** Social and professional characteristic features of the respondents

Social & professional variables	Measurement	Value range	Frequency	Median	Mean	std.dev.
Gender	Binary	Male = 0 Female = 1	38 (55%) 31 (45%)	0	0.45	0.50
Age group	Ordinal	“26–30” = 2 “31–40” = 3 “41–50” = 4 “51–60” = 5 “>60” = 6	3 (4%) 13 (19%) 30 (43%) 22 (32%) 1 (1%)	4	4.07	0.86
Country of origin	Nominal	Viet Nam = 1 Elsewhere = 0	53 (77%) 16 (23%)			
Area of work: Program	Binary	No = 0 Yes = 1	23 (33%) 46 (67%)	1	0.67	0.47
Area of work: Policy	Binary	No = 0 Yes = 1	41 (59%) 28 (41%)	0	0.41	0.49
Area of work: Management	Binary	No = 0 Yes = 1	29 (42%) 40 (58%)	1	0.58	0.49
Area of work: Monitoring & Evaluation	Binary	No = 0 Yes = 1	31 (45%) 38 (55%)	1	0.55	0.50
Years of work experience in HIV field	Scale	From 3 to 30 years	–	14	14.6	6.19
Involved in decision-making	Binary	No = 0 Yes = 1	6 (9%) 63 (91%)	1	0.91	0.28
Responsible for decision-making	Binary	No = 0 Yes = 1	24 (35%) 45 (65%)	1	0.65	0.48

The self-explicated ranking of the criteria based on all responses shows that effectiveness is the most important criterion for respondents with 43% of respondents ranking this criterion very high. Feasibility is the next highest ranked criterion followed by sustainability, cost-effectiveness and treatment to prevention spending ratio. When responses are disaggregated by agency, effectiveness remains the most important criterion and treatment to prevention spending ratio remains the least important criterion. Most civil society respondents (60%) and development partner respondents (48%) select effectiveness as their most important criterion, while government respondents are equally split on feasibility and effectiveness as their top criteria (27 and 27%). The results of the self-explicated ranking are presented in Table 3.

**Table 3:** Self-explicated rankings of criteria for prioritizing the HIV response

	Most important			Least Important		Mode	Median	Mean	std.dev.
Criteria	1	2	3	4	5				
All Stakeholders									
Effectiveness	43%	29%	16%	7%	4%	1	2	2.00	1.007
Cost-effectiveness	14%	20%	13%	38%	14%	4	4	3.17	1.221
Sustainability	14%	19%	28%	28%	12%	3	3	3.03	1.133
Treatment/Prevention	6%	4%	17%	10%	62%	5	5	4.19	1.246
Feasibility	22%	28%	26%	17%	7%	2	3	2.61	1.102
Development Partners									
Effectiveness	48%	26%	9%	13%	4%	1	2	2.00	1.103
Cost-effectiveness	13%	22%	13%	30%	22%	4	4	3.26	1.251
Sustainability	4%	26%	26%	30%	13%	4	3	3.22	1.016
Treatment/Prevention	4%	4%	30%	9%	52%	5	5	4.00	1.180
Feasibility	30%	22%	22%	17%	9%	1	2	2.52	1.187
Government									
Effectiveness	27%	38%	31%	0%	4%	2	2	2.15	0.835
Cost-effectiveness	15%	8%	12%	58%	8%	4	4	3.35	1.172
Sustainability	23%	15%	27%	27%	8%	3	3	2.81	1.190
Treatment/Prevention	8%	4%	8%	8%	73%	5	5	4.35	1.304
Feasibility	27%	35%	23%	8%	8%	2	2	2.35	1.021
Civil Society									
Effectiveness	60%	20%	5%	10%	5%	1	1	1.80	1.053
Cost-effectiveness	15%	35%	15%	20%	15%	2	2	2.85	1.165
Sustainability	15%	15%	30%	25%	15%	3	3	3.10	1.155
Treatment/Prevention	5%	5%	15%	15%	60%	5	5	4.20	1.207
Feasibility	5%	25%	35%	30%	5%	3	3	3.05	0.925

Results of the logit regression of the main effect model, after removing the inconsistent responses, are shown in Table 4. The results for the main effect model show that overall the feasibility criterion is the most important to the respondents when choosing a hypothetical HIV program, followed by sustainability, treatment to prevention spending ratio, and effectiveness. The coefficient of cost-effectiveness in the main-effects model is not statistically significant. However, the main effect model does not account for the social and professional characteristics of the respondents. The influence of these characteristics can be seen in the reduced model with interactions.

**Table 4:** Result of the discrete choice experiment on criteria for prioritizing the HIV response, main effect model together with interactions

Dependent variable (0 = if the basic profile is chosen; 1 = if an alternative profile is chosen)				
	Main Effect Model		Model w/ Interactions	
Criteria	β	std.err.	β	std.err.
Independent variables (coding under variables)				
Δ Effectiveness	0.713*	0.328	5.482*	1.577
0 = no change, remains 4 million years of life saved				
1 = increases, 5 instead of 4 million years of life saved				
Δ Cost-effectiveness	0.240	0.296	0.184	0.317
0 = no change, remains $6 return for every $1 invested				
-1 = decreases, $5 instead of $6 return for every $1 invested				
Δ Sustainability	1.309*	0.336	1.377*	0.583
0 = no change, spending increases and then decreases				
-1 = changes to spending constantly increases				
Δ Treatment/Prevention	0.780*	0.312	1.486*	0.466
0 = no change, greater investment in prevention				
-1 = changes to greater investment in treatment				
Δ Feasibility	1.980*	0.327	3.190*	0.616
0 = no change, remains low feasibility				
1 = increases, high feasibility instead of low feasibility				
Constant	0.887	0.669	0.724	0.709
Interaction Terms (w/ Δ Effectiveness)				
works in programming			−1.723*	0.563
involved in decision-making			−3.623*	1.457
Interaction Terms (w/ Δ Sustainability)				
works in management			1.199*	0.539
responsible for decisions			−1.204*	0.590
Interaction Terms (w/ Δ Treatment/Prevention)				
works in management			−1.156*	0.512
Interaction Terms (w/ Δ Feasibility)				
responsible for decisions			−1.412*	0.633
rho	0.491		0.522	
pseudo r-squared	0.190		0.245	

*ρ < .05

Specifically, the reduced model with interactions shows that several interactions of criteria with social and professional characteristics of the respondents have a significant effect (Table 4). The backward stepwise regression finds six interaction terms that are statistically significant. Two interaction terms are with the effectiveness criterion. Those interactions are with respondents who are currently working, or have worked, in programming and respondents who are currently, or have been, involved in decision-making. Two other interaction terms are with the sustainability criterion. Those interactions are with respondents who are currently working, or have worked, in management and respondents who are currently, or have been, responsible for decisions. One interaction is with the criterion of the ratio of treatment to prevention spending. That interaction is with respondents who are currently working, or have worked, in management. Another interaction term is with the feasibility criterion. That interaction is with respondents who are currently, or have been, responsible for decisions. All interactions, except for the interaction of sustainability with working in management, have an overall negative effect on the ranking of their respective criteria.

DCE analysis results by agency show that development partners, civil society, as well as government respondents all rank the feasibility criterion high. Sustainability, the ratio of treatment to prevention spending, and feasibility are in the top three criteria of both civil society and government respondents. Effectiveness is highly ranked by the development partners only. The coefficients of other criteria do not carry sufficient statistical weight to confidently say how they are ranked. Table 5 presents the ranking of criteria per each agency.

**Table 5:** Result of discrete choice experiment regarding criteria for prioritizing the HIV response, main effects model, disaggregated by agency

Dependent variable (0 = if the basic profile is chosen; 1 = if an alternative profile is chosen)						
	Dvlp. Partners		Government		Civil Society	
Criteria	β	std.err.	β	std.err.	β	std.err.
Independent variables (coding under variables)						
Δ Effectiveness	2.021*	0.776	0.848	0.515	−0.356	0.632
0 = no change, remains 4 million years of life saved						
1 = increases, 5 instead of 4 million years of life saved						
Δ Cost-effectiveness	0.081	0.729	0.413	0.467	−0.061	0.545
0 = no change, remains $6 return for every $1 invested						
-1 = decreases, $5 instead of $6 return for every $1 invested						
Δ Sustainability	0.028	0.736	1.561*	0.533	2.125*	0.685
0 = no change, spending increases and then decreases						
-1 = changes to spending constantly increases						
Δ Treatment/Prevention	−0.277	0.569	1.190*	0.503	1.140*	0.640
0 = no change, greater investment in prevention						
-1 = changes to greater investment in treatment						
Δ Feasibility	3.113*	0.828	1.514*	0.492	2.202*	0.641
0 = no change, remains low feasibility						
1 = increases, high feasibility instead of low feasibility						
Constant	−1.073	1.465	1.414	1.173	1.355	1.345
Number of observations	147		182		140	
Number of groups	21		26		20	
Log likelihood function	−60.46		−90.12		−66.92	
Wald chi2	21.63		27.93		22.18	
prob > chi2	0.001		0.000		0.001	

*ρ < .05

## Discussion

All else being equal, participants prefer a program that is most feasible, front-loaded for sustainability, has a higher proportion of investment on prevention, saves more lives and prevents more infections, and is more cost-effective although this latter criterion does not show a statistically significant effect on the choices in the DCE.

The self-explicated ranking of criteria finds the “effectiveness” criterion to be the highest ranked criterion by respondents. This is in keeping with previous studies in rating importance of criteria in Asia that also found effectiveness to be the most important criterion for prioritizing interventions in the HIV response [15, 25]. A systematic review of criteria in priority setting of HIV and health care also found effectiveness to be among the highest cited criteria in the literature [33, 40]. Similar to these previous studies, during the self-explicated portion of this study, criteria were presented to participants as concepts without quantification, for example in terms of lives saved. However, during the DCE in this study, the effectiveness criterion dropped to fourth place according to its ranked importance to the respondents. This difference between the self-explicated ranking and DCE ranking may suggest the presence of social desirability bias in the self-explicated ranking. That is, when confronted with the general notion of a criterion such as effectiveness or sustainability, respondents may rely on their principles to determine its importance. However, when given more specific trade-off tasks during the DCE, for example to compare programs with nominal gains in lives saved at lower feasibility, the respondents may rely on their professional expertise to make their decisions. This phenomenon is further reinforced by the effectiveness criterion being consistently ranked highest by respondents from all different agency affiliations during self-explicated ranking, suggesting that the respondents’ agency affiliations do not influence their decision. However, during the DCE only development partners ranked the effectiveness criterion high. Furthermore, DCE results show that the interaction of program effectiveness with professional characteristics of “working in programming” and “being involved in decision-making” significantly lower the ranking of the effectiveness criterion, suggesting that when the same respondents are asked to rank the program options, the ones with professional responsibilities in delivering program recommendations reconsider their priorities and lower their ranking of program effectiveness as a criterion vis-à-vis other criteria.

Another finding from the interaction results is the difference in ranking of criteria by respondents who have worked in management of HIV programs. Compared to the average response, those who worked in management rate prevention/treatment ratio lower and rate sustainability of programs higher. These results indicate some theoretical consideration in the decision-making of program managers based on financial models [41], giving a longer term view of the sustainability of the program even though it requires a large upfront investment. The program managers also consider a lower investment in prevention to offset a higher investment in treatment, which also indicates a theoretical approach given the mathematical models that suggest universal test and treatment programs could drive HIV eradication [42, 43] even though a more pragmatic view based on empirical evidence suggests many barriers in the cascade of care to link and retain patients in treatment [44–46].

The interaction terms also show that those responsible for decisions rate the feasibility and sustainability criteria lower than the average respondent. This is consistent with the traditional model of public service governance where decision-makers are concerned with the outcomes of the programs they choose, and feasibility and sustainability are considerations for actors at different levels of their hierarchical organization [47]. Program planning is often sequenced from objective analysis, to activity planning, and ending with analysis of risks, with outcome results considered in the initial stages, and feasibility and sustainability considered in the later stages [48]. This planning process is likely to have contributed to a program option that reflects more strongly the effectiveness criterion that is considered earlier in the process than other relevant criteria considered later in the planning process. Other iterative models of planning or greater involvement of stakeholders at all stages of planning may be needed to ensure relevant criteria are considered at appropriate decision points. This perspective is gaining traction in the recognition that the problems and solutions of public health cannot be solely owned by the government but require collaboration and engagement of multiple stakeholders [49].

Feasibility and sustainability are ranked highly both in the DCE as well as the self-explicated ranking. Although respondents from the civil society and government differ in the ordering of these two criteria in the DCE, their responses indicate that they agree the two criteria are the most important for prioritizing HIV programs. This result can be useful in the advocacy for greater involvement of the civil society in the priority setting process together with the government, since it brings to light that there are more points of agreement than differences between the two groups. While development partners agree with the government and civil society on the importance of the feasibility criterion, they consider the program effectiveness as their second most important criterion. Future priority setting processes may consider the level of importance of these criteria to different stakeholders, and develop program options that cater to their values. The transparency in options and weight of criteria according to different stakeholders will facilitate and focus discussions around trade-offs that need to be made and between whom.

The coefficient for the cost-effectiveness criterion was not significant in the regression analysis. In other words, the difference between the preference weight of the more cost-effective program and the less cost-effective program was not statistically significant. There could be two reasons for this: either we are unable to estimate the coefficients efficiently with the model used (e.g. too small difference between the levels of that criterion with no significant effect on the choice), or there is too much heterogeneity in the preferences for the cost-effectiveness criterion. The cost-effectiveness criterion however ranks low in self-explicated rankings, across all groups. This seems surprising if we consider the extensive use of the criterion for prioritization in healthcare, and guidelines developed for cost-effectiveness analysis of healthcare programs [40, 50, 51]. However, a comparable phenomenon is observed in some Central and Eastern European countries with similar political-economy histories to Viet Nam, where cost-effectiveness is considered a “soft” criteria in healthcare priority setting [52, 53].

Between 2006 and 2010, the national HIV programs resulted in an estimated 401,600 fewer disease adjusted life years (DALY) at an estimated cost of $248 for each DALY averted [54]. The DCE results in this study indicate a preference for 5 million years of life saved from death and disease between 2016 and 2030, and $315 for each DALY averted. These findings demonstrate that the stakeholders in this study prefer greater effectiveness of HIV programs in the future, but do not expect much change in the cost-effectiveness of the HIV programs. The 2014 national investment case [5] used two criteria of effectiveness and cost-effectiveness to rank several modeled HIV programs. Similar to findings of this study, effectiveness was prioritized over cost-effectives in the ranking of the choices. The investment case also considered a scenario where resource needs increase over time, as the “worst-case scenario”, which is consistent with results of the DCE in this study on the sustainability criterion. The currently implemented national HIV program can also indicate the ratio of treatment to prevention spending. Recently prevention has accounted for close to 25% of funds of the HIV response, indicating that the national plan leans toward lower prevention spending, whereas findings of this study indicate a preference for greater prevention spending.

Overall, the DCE method is shown to be effective and feasible in establishing priorities in Viet Nam. It provides additional important information beyond what the self-explicated ranking of criteria provides, such as the comparative importance of one criterion against another. It also explains the direction of criteria that is preferred by the respondents. For example, whether they prefer greater prevention or greater treatment in the prevention/treatment ratio criterion. However, the DCE also requires a large number of respondents to make reliable estimates, which may not be feasible in countries with a small program and few people involved in decision making at the central level to respond to the questionnaire. With few respondents, the number of criteria to be considered may be limited, jeopardizing the validity of the results.

This study has several strengths and limitations that need to be acknowledged. The strength of this study is in considering a broad set of criteria relevant to the country, and ranking them with experimental and self-explicated methods for improved accuracy and precision. Our study also involves multiple groups of stakeholders representing the different perspectives of those who should be involved in prioritizing Viet Nam’s HIV response. This study has fewer participants in the DCE than other similar studies. Although we tried to reach a maximum number of actors with experience or expertise in decision-making on HIV programs, the HIV space in Viet Nam is ultimately limited by the size of the epidemic and response. Given the limited number of potential participants, and the desire to minimize the cognitive load of the DCE, a limited number of criteria are considered for prioritization from the full set.

This study occurs at a transition period in Viet Nam, as official development assistance to the HIV response is being reduced, and greater domestic investments including social health insurance are being mobilized to cover the gap left by the donors. The stakeholders’ ranking of the criteria for prioritizing HIV programs presented here may be a reflection of the current context in Viet Nam, which could change in the future.

## Conclusions

Findings of this study show that there are greater similarities between the ranking of criteria by government and civil society than there are differences. The process and results in elicitation of the importance of the criteria can inform future policy dialogues between the stakeholders to find common grounds in priority setting. The results also highlight the need to reconsider the classical hierarchical models of planning in Viet Nam, and utilize innovative models of planning that allow inputs of informed stakeholders at relevant stages of the HIV program planning process. The results may also be useful for other developing countries in a transition period to visit or revisit the criteria used to prioritize their HIV programs. In donor supported countries, the transparent process of eliciting criteria for HIV program prioritization can be an additional requirement for funding proposals that demonstrates wide stakeholder consultation, and evidence-based planning and prioritization.

As Viet Nam moves closer to becoming an upper-middle-income country, and donors transition away from direct support of the HIV response, the importance of certain criteria for prioritizing the HIV program package will need to be re-evaluated. Cost-effectiveness is one criterion used prominently in the past investment case analysis of Viet Nam’s HIV response, but ranks lower in this study. Going forward, this criterion should again be considered centrally once programs transition from donor support to domestic financing, and standalone HIV programs integrate back into the general healthcare system, and evidence becomes available on the cost and effectiveness of this newly integrated program structure.

## Additional files

Additional file 1: Contains the survey questionnaire in English. (DOCX 348 kb)

Additional file 2: Contains the survey questionnaire in Vietnamese. (DOCX 481 kb)

## Abbreviations

AEM: AIDS epidemic model
AIDS: Acquired immunodeficiency syndrome
AusAID: Australian Agency for International Development
DALY: Disease adjusted life years
DCE: Discrete choice experiment
DFID: UK Department for International Development
Dvlp. Partners: Development partners
GFATM: The global fund to fight AIDS, tuberculosis and malaria
GIPA: Greater involvement of people living with HIV/AIDS
HIV: Human immunodeficiency virus
KFF: Kaiser Family Foundation
PEPFAR: The U.S. President’s Emergency Plan for AIDS Relief
UNAIDS: The Joint United Nations Programme on HIV/AIDS
