# Supplementary material for: Criteria for prioritization of HIV programs in Viet Nam: a discrete choice experiment
Source: BMC Health Serv Res. 2017 Nov 13;17:719. doi: 10.1186/s12913-017-2679-0 (PMC5683339; doi:10.1186/s12913-017-2679-0)
Supplement: Supplementary file 1 — Contains the survey questionnaire in English. (DOCX 348 kb) [file 12913_2017_2679_MOESM1_ESM.docx]

Top of Form

# Survey on Criteria for HIV Program Selection in Viet Nam

Welcome to the survey on Criteria for HIV Program Selection in Viet Nam. This survey is anonymous and is carried out for academic purposes only. You may exit and clear the survey at any time while answering the questions, should you feel uncomfortable.

If you agree to start, please press the "NEXT" button.


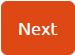


Survey on Priority Criteria in the Viet Nam HIV Response

Please tell us a bit about yourself:

**Your Gender:**

☐ Male ☐ Female

**Your Age:**

☐ <22 ☐ 22-25 ☐ 26-30 ☐ 31-40 ☐ 41-50 ☐ 51-60 ☐ >60

**Your Country of Origin:**

☐ Việt Nam ☐ Other: ___________________

**Your organization:**

☐ Government

☐ United Nations

☐ Donor Agency

☐ Research/Academic Institution

☐ Civil Society/Community Organization

☐ Other: ___________________

**Your area of work (***you can choose multiple options***):**

☐ Programs

☐ Policy

☐ Management

☐ Monitoring & Evaluation

☐ Other: ___________________

**Years of experience working in HIV response:** ______________

**Have you been involved in making decisions on HIV programs?**

☐ Yes ☐ No

**Have you been responsible for making decisions about HIV programs?**

☐ Yes ☐ No


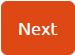


Survey on Priority Criteria in the Viet Nam HIV Response

Top of Form

Imagine a situation when you have to decide on a suitable program for HIV response in Viet Nam, given a fixed budget. You could base your decision on several criteria:

- **Effectiveness**: Years of life saved from death or disease
- **Sustainability**: Maintaining or reducing resource needs
- **Prevention/Treatment Spending Ratio**: Spending on prevention versus spending on treatment
- **Cost-Effectiveness**: Economic returns of program versus cost of program
- **Feasibility**: Likelihood of achieving the expected scale and rate of increase in coverage set by the program

Please order these criteria in terms of their importance to you if you had to make such a decision:

*Your most important criterion should be on the top right, moving through to your least important criterion.*

**Your choices Your ranking**

- Effectiveness
- Sustainability
- Prevention/Treatment Spending Ratio
- Cost-Effectiveness
- Feasibility


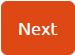


Survey on Priority Criteria in the Viet Nam HIV Response

In the following pages you will be presented with seven scenarios. In each scenario you will be asked to choose between two programs for HIV response in Viet Nam. All programs carry the same cost of $80 Million USD. The two programs presented to you in each scenario are similar in every way, except for those attributes that are highlighted.

The purpose of this survey is not to find the best programs. Therefore, there is no wrong answer to the choices you make. Please use your values to choose between the programs presented in each scenario.


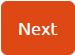


Survey on Priority Criteria in the Viet Nam HIV Response

Imagine a situation when you have to decide on a suitable program for HIV response in Viet Nam.

**Which of these programs would you choose?** Both programs have the same cost of $80 Million USD. To make it easier for you, we have highlighted the characteristics that differ between the two programs.


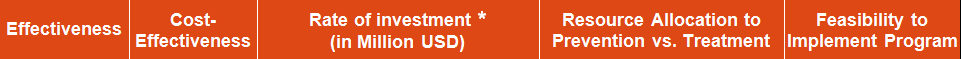


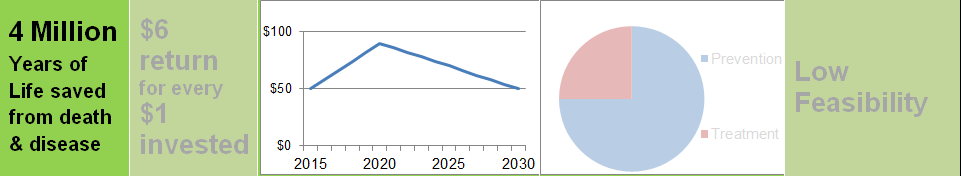


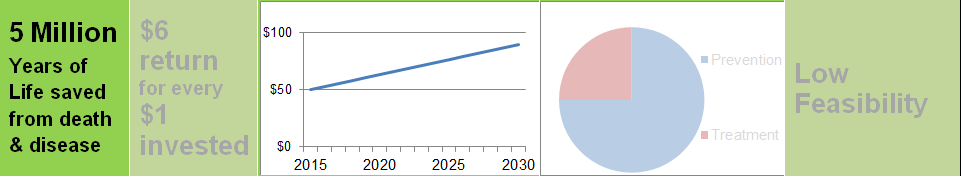


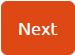


Survey on Priority Criteria in the Viet Nam HIV Response

Imagine a situation when you have to decide on a suitable program for HIV response in Viet Nam.

**Which of these programs would you choose?** Both programs have the same cost of $80 Million USD. To make it easier for you, we have highlighted the characteristics that differ between the two programs.


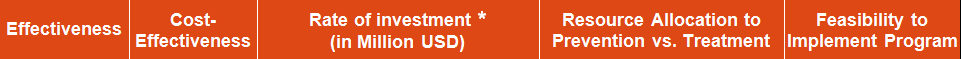


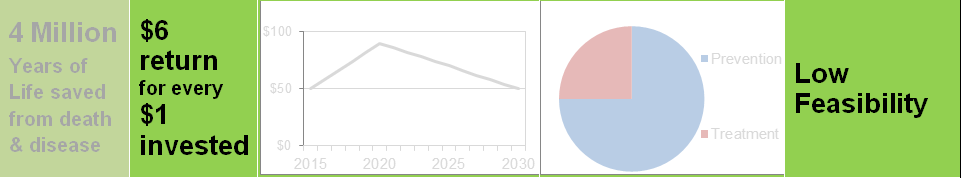


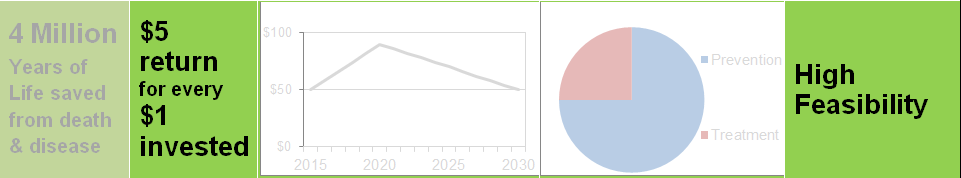


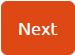


Survey on Priority Criteria in the Viet Nam HIV Response

Imagine a situation when you have to decide on a suitable program for HIV response in Viet Nam.

**Which of these programs would you choose?** Both programs have the same cost of $80 Million USD. To make it easier for you, we have highlighted the characteristics that differ between the two programs.


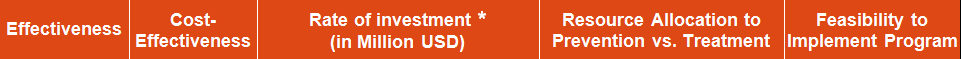


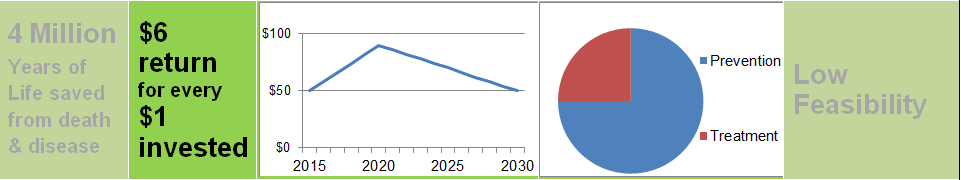


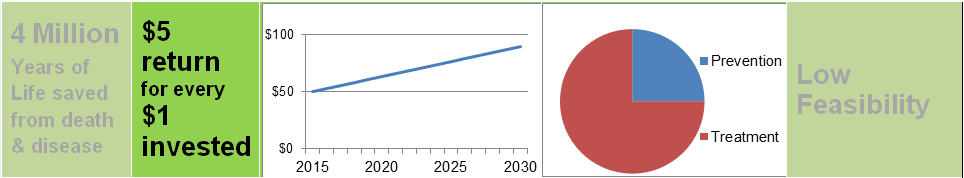


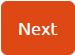


Survey on Priority Criteria in the Viet Nam HIV Response

Imagine a situation when you have to decide on a suitable program for HIV response in Viet Nam.

**Which of these programs would you choose?** Both programs have the same cost of $80 Million USD. To make it easier for you, we have highlighted the characteristics that differ between the two programs.


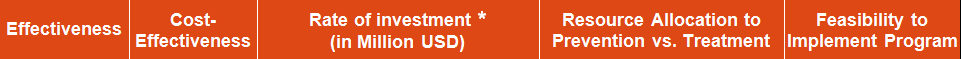


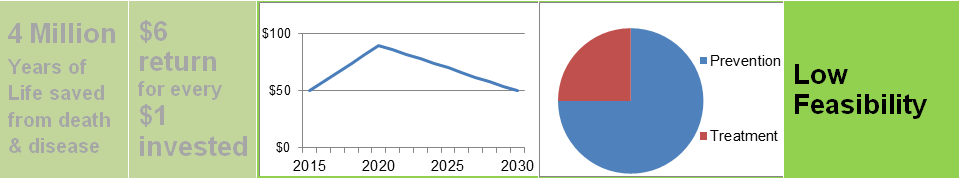


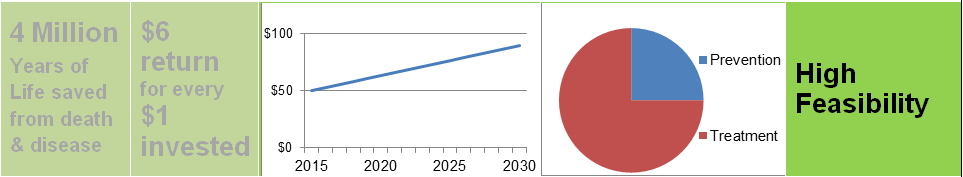


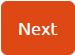


Survey on Priority Criteria in the Viet Nam HIV Response

Imagine a situation when you have to decide on a suitable program for HIV response in Viet Nam.

**Which of these programs would you choose?** Both programs have the same cost of $80 Million USD. To make it easier for you, we have highlighted the characteristics that differ between the two programs.


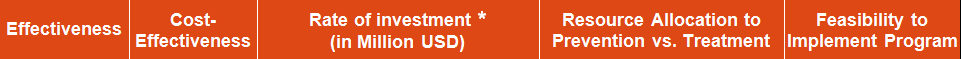


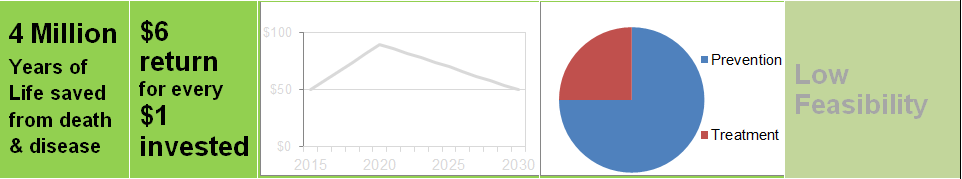


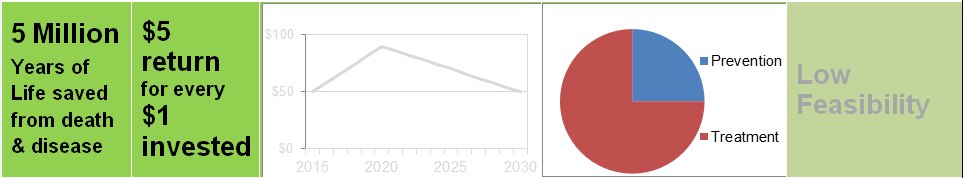


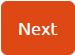


Survey on Priority Criteria in the Viet Nam HIV Response

Imagine a situation when you have to decide on a suitable program for HIV response in Viet Nam.

**Which of these programs would you choose?** Both programs have the same cost of $80 Million USD. To make it easier for you, we have highlighted the characteristics that differ between the two programs.


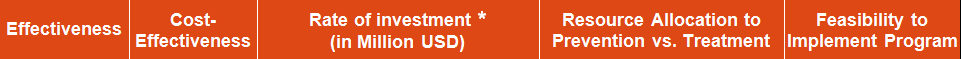


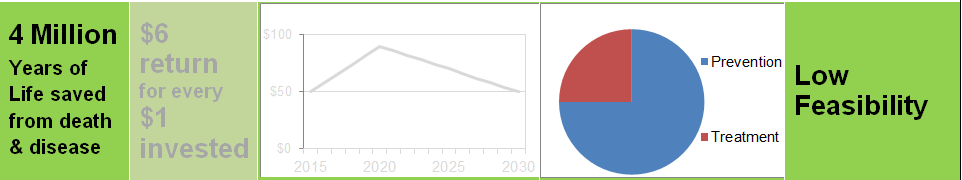


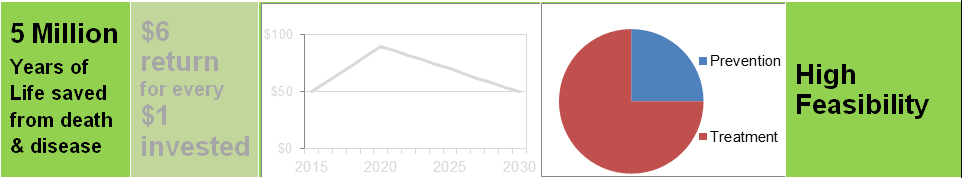


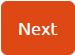


Survey on Priority Criteria in the Viet Nam HIV Response

Imagine a situation when you have to decide on a suitable program for HIV response in Viet Nam.

**Which of these programs would you choose?** Both programs have the same cost of $80 Million USD. To make it easier for you, we have highlighted the characteristics that differ between the two programs.


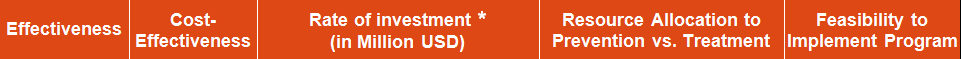


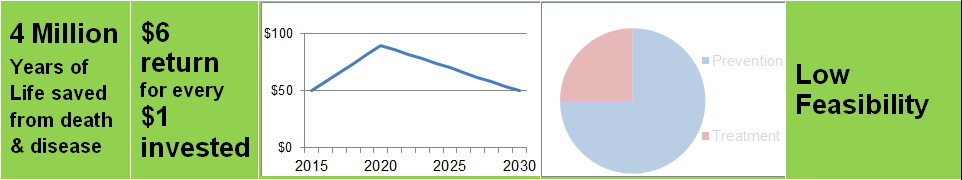


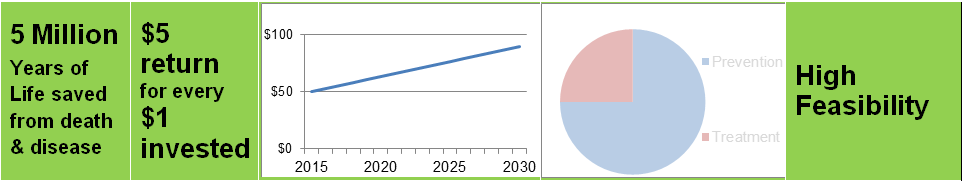


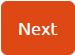


Survey on Priority Criteria in the Viet Nam HIV Response

Thank you for participating in this survey. If you have any questions, please email: [a.safarnejad@student.maastrichtuniversity.nl](mailto:a.safarnejad@student.maastrichtuniversity.nl)
