# Supplementary material for: Criteria for prioritization of HIV programs in Viet Nam: a discrete choice experiment
Source: BMC Health Serv Res. 2017 Nov 13;17:719. doi: 10.1186/s12913-017-2679-0 (PMC5683339; doi:10.1186/s12913-017-2679-0)
Supplement: Supplementary file 2 — Contains the survey questionnaire in Vietnamese. (DOCX 481 kb) [file 12913_2017_2679_MOESM2_ESM.docx]

Top of Form

# Khảo sát các tiêu chí lựa chọn chương trình phòng, chống HIV/AIDS tại Việt Nam

Chào mừng bạn đến cuộc khảo sát về tiêu chí lựa chọn Chương trình HIV/AIDS tại Việt Nam. Cuộc khảo sát này không thu thập thông tin cán nhân của người tham gia trả lời câu hỏi khảo sát và khảo sát này được thực hiện với mục đích học tập. Bạn có thể ngừng trả lời các câu hỏi bất cứ lúc nào nếu bạn sẽ cảm thấy không thoải mái.

Nếu bạn đồng ý tham gia khảo sát này, xin vùi lòng nhấn nút “Next” dưới đây.


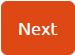


Khảo sát các tiêu chí lựa chọn chương trình phòng, chống HIV/AIDS tại Việt NamTop of Form

Vui lòng cho chúng tôi biết về bạn:

**Giới tính:**

☐ Nam ☐ Nữ

**Tuổi:**

☐ <22 ☐ 22-25 ☐ 26-30 ☐ 31-40 ☐ 41-50 ☐ 51-60 ☐ >60

**Bạn là người nước nào:**

☐ Việt Nam ☐ Khác: ___________________

**Cơ quan của anh chị thuộc:**

☐ Cơ quan nhà nước

☐ Cơ quan thuộc Liên hợp quốc

☐ Các nhà tài trợ

☐ Các viện nghiên cứu

☐ Các tổ chức xã hội dân sự/cộng đồng

☐ Khác: ___________________

**Bạn làm trong lĩnh vực gì**(*Bạn có thể chọn nhiều câu trả lời*)**:**

☐ Triển khai chương trình

☐ Xây dựng chính sách

☐ Quản lý

☐ Theo dõi và đánh giá

☐ Khác: ___________________

**Số năm kinh nghiệm làm việc trong lĩnh vực phòng, chống HIV/AIDS: _________________**

**Bạn đã từng được tham gia ra quyết định về lựa chọn chính sách, giải pháp, kế hoạch cho hoạt động phòng, chống HIV/AIDS cấp quốc gia hoặc địa phương?**

☐ Có ☐ Không

**Bạn đã bao giờ chịu trách nhiệm ra quyết định về lựa chọn chính sách, giải pháp, kế hoạch cho hoạt động phòng, chống HIV/AIDS cấp quốc gia hoặc địa phương mình?**

☐ Có ☐ Không

Khảo sát các tiêu chí lựa chọn chương trình phòng, chống HIV/AIDS tại Việt NamTop of Form

Giả thuyết có một tình huống bạn phải quyết định lựa chọn một giải pháp phù hợp để ứng phó với dịch HIV/AIDS tại Việt Nam, giả định ngân sách cho phòng, chống HIV/AIDS không thay đổi. Bạn căn cứ vào tiêu chí nào sau đây để ra quyết định:

- **Tính hiệu quả**: Số người được dự phòng không bị nhiễm HIV hoặc tử vong do AIDS
- **Tính bền vững**: Duy trì và giảm nhu cầu nguồn lực
- **Tỷ lệ đầu tư cho Dự phòng/Điều trị**: Chi tiêu cho dự phòng so với chi tiêu cho điều trị
- **Chi phí – Hiệu quả**: Hiệu quả kinh tế của chương trình mang lại so với chi phí đầu tư cho chương trình.
- **Tính khả thi:** Khả năng thực hiện được các mục tiêu đặt ra. Ví dụ chỉ có 45% người nhiễm HIV vào năm 2015, liệu có khả thi để đạt được mục tiêu 90% người nhiễm HIV được điều trị ARV vào 2020.

Vui lòng sắp xếp các tiêu chí này theo thứ tự về tầm quan trọng nếu bạn phải quyết định vấn đề đầu tư cho chương trình phòng, chống HIV/AIDS:

*tiêu chí quan trọng nhất bạn đặt  phía trên bên phải, di chuyển thông qua các tiêu chí quan trọng tiếp theo.*

**Your choices Your ranking**

- Tính hiệu quả
- Tính bền vững
- Tỷ lệ đầu tư cho Dự phòng/Điều trị
- Chi phí – Hiệu quả
- Tính khả thi


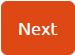


Khảo sát các tiêu chí lựa chọn chương trình phòng, chống HIV/AIDS tại Việt NamTop of Form

Những trang tiếp theo sẽ có **7** kịch bản về đầu tư cho phòng, chống HIV/AIDS tại Việt Nam. Mỗi một kịch bản bạn được yêu cầu chọn 2 tiêu chí để quyết định giải pháp cho Hoạt động phòng, chống HIV/AIDS. Tất cả chương trình đều đầu tư mức trung bình 80 triệu đô la/năm trong giai đoạn 2015-2030. Hai cách thức thực hiện trong mỗi kịch bản là tương tự nhau từng tiêu chí, ngoại trừ những tiêu chí có hình ảnh sáng hơn.

Mục đích của điều tra này là không để tìm ra chương trình tốt nhất. Hơn nữa, không có câu trả lời sai về cách bạn đã chọn. Vui lòng dựa vào quan điểm của bạn để chọn một trong 2 chương trình giới thiệu dưới đây trong mỗi kịch bản.


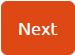


Khảo sát các tiêu chí lựa chọn chương trình phòng, chống HIV/AIDS tại Việt NamTop of Form

Hãy hình dung một tình huống khi bạn phải quyết định một chương trình bền vững để đối phó với HIV tại Việt Nam.

**Những tiêu chí nào sau đây bạn sẽ chọn?** Cả 2 giải pháp được đề xuất dưới đây đều có mức đầu tư trung bình 80 triệu đô la mỗi năm. Để dễ cho việc lựa chọn, chúng tôi đã để hình ảnh sáng hơn cho sự khác nhau giữa 2 giải pháp.


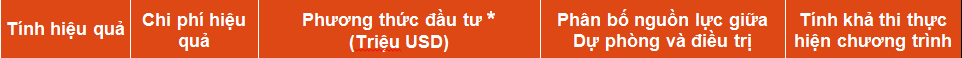


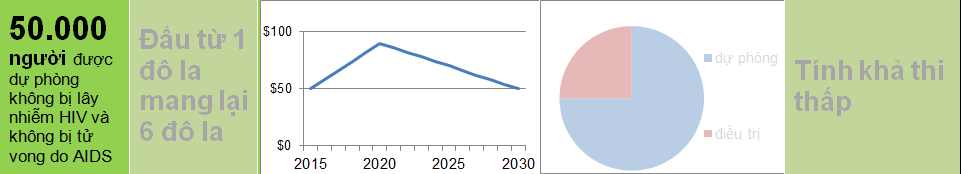


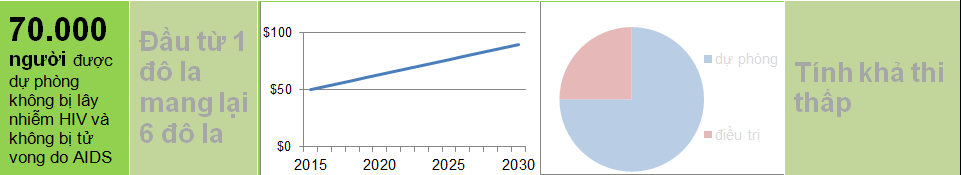


* 1. Đầu tư tăng nhanh trong 5 năm đầu, sau đó giảm dần qua các năm.

* 2. Đầu tư tăng dần qua các năm.


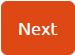


Khảo sát các tiêu chí lựa chọn chương trình phòng, chống HIV/AIDS tại Việt NamTop of Form

Hãy hình dung một tình huống khi bạn phải quyết định một chương trình bền vững để đối phó với HIV tại Việt Nam.

**Những tiêu chí nào sau đây bạn sẽ chọn?** Cả 2 giải pháp được đề xuất dưới đây đều có mức đầu tư trung bình 80 triệu đô la mỗi năm. Để dễ cho việc lựa chọn, chúng tôi đã để hình ảnh sáng hơn cho sự khác nhau giữa 2 giải pháp.


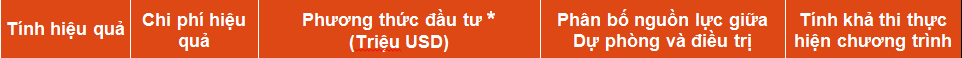


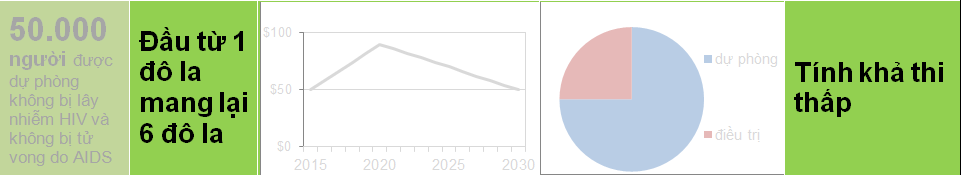


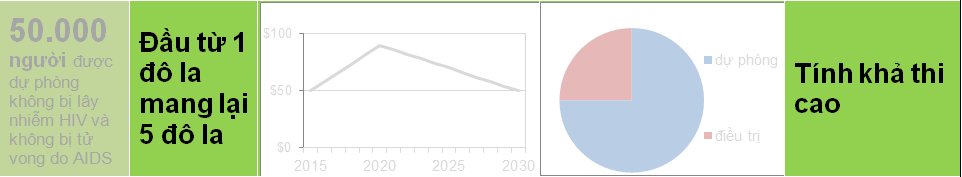


1. Đầu tư tăng nhanh trong 5 năm đầu, sau đó giảm dần qua các năm


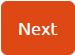


Khảo sát các tiêu chí lựa chọn chương trình phòng, chống HIV/AIDS tại Việt NamTop of Form

Hãy hình dung một tình huống khi bạn phải quyết định một chương trình bền vững để đối phó với HIV tại Việt Nam.

**Những tiêu chí nào sau đây bạn sẽ chọn?** Cả 2 giải pháp được đề xuất dưới đây đều có mức đầu tư trung bình 80 triệu đô la mỗi năm. Để dễ cho việc lựa chọn, chúng tôi đã để hình ảnh sáng hơn cho sự khác nhau giữa 2 giải pháp.


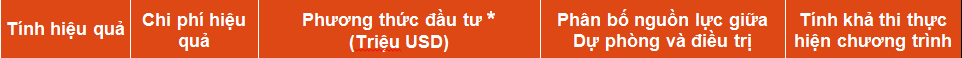


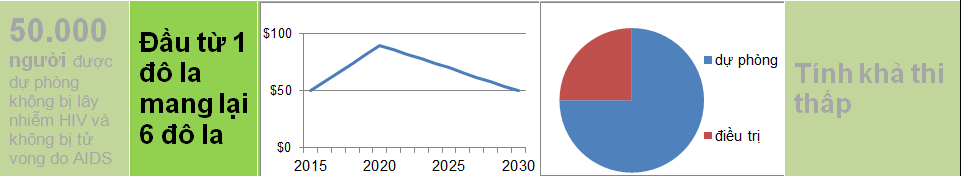


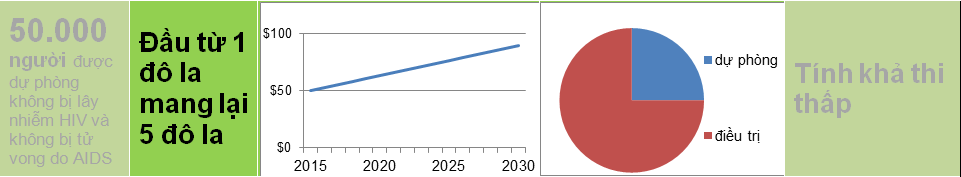


* 1. Đầu tư tăng nhanh trong 5 năm đầu, sau đó giảm dần qua các năm.

* 2. Đầu tư tăng dần qua các năm


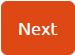


Khảo sát các tiêu chí lựa chọn chương trình phòng, chống HIV/AIDS tại Việt NamTop of Form

Hãy hình dung một tình huống khi bạn phải quyết định một chương trình bền vững để đối phó với HIV tại Việt Nam.

**Những tiêu chí nào sau đây bạn sẽ chọn?** Cả 2 giải pháp được đề xuất dưới đây đều có mức đầu tư trung bình 80 triệu đô la mỗi năm. Để dễ cho việc lựa chọn, chúng tôi đã để hình ảnh sáng hơn cho sự khác nhau giữa 2 giải pháp.


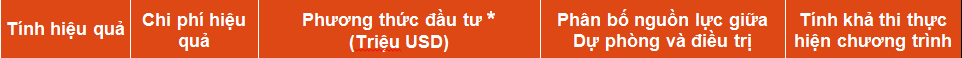


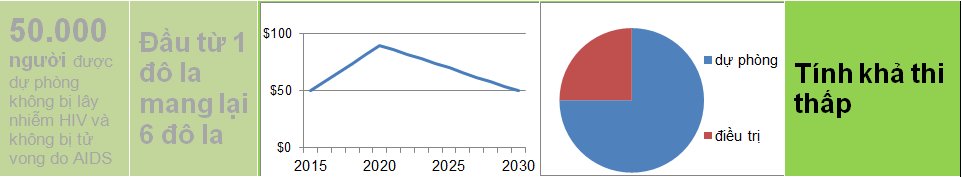


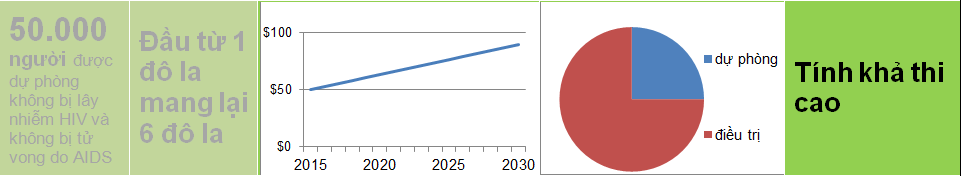


* 1. Đầu tư tăng nhanh trong 5 năm đầu, sau đó giảm dần qua các năm.

* 2. Đầu tư tăng dần qua các năm


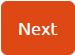


Khảo sát các tiêu chí lựa chọn chương trình phòng, chống HIV/AIDS tại Việt NamTop of Form

Hãy hình dung một tình huống khi bạn phải quyết định một chương trình bền vững để đối phó với HIV tại Việt Nam.

**Những tiêu chí nào sau đây bạn sẽ chọn?** Cả 2 giải pháp được đề xuất dưới đây đều có mức đầu tư trung bình 80 triệu đô la mỗi năm. Để dễ cho việc lựa chọn, chúng tôi đã để hình ảnh sáng hơn cho sự khác nhau giữa 2 giải pháp.


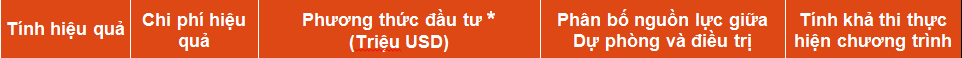


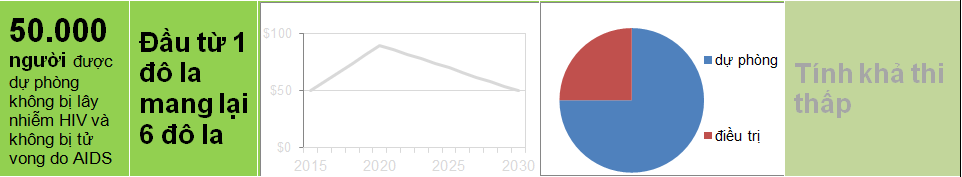


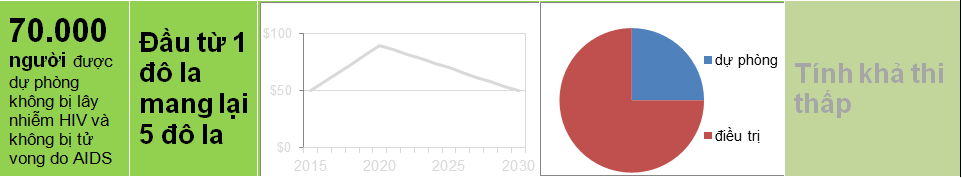


  * 1. Đầu tư tăng nhanh trong 5 năm đầu, sau đó giảm dần qua các năm.


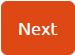


Khảo sát các tiêu chí lựa chọn chương trình phòng, chống HIV/AIDS tại Việt NamTop of Form

Hãy hình dung một tình huống khi bạn phải quyết định một chương trình bền vững để đối phó với HIV tại Việt Nam.

**Những tiêu chí nào sau đây bạn sẽ chọn?** Cả 2 giải pháp được đề xuất dưới đây đều có mức đầu tư trung bình 80 triệu đô la mỗi năm. Để dễ cho việc lựa chọn, chúng tôi đã để hình ảnh sáng hơn cho sự khác nhau giữa 2 giải pháp.


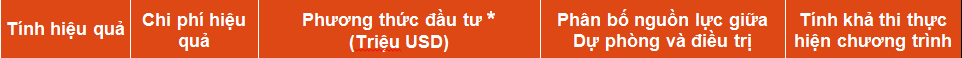


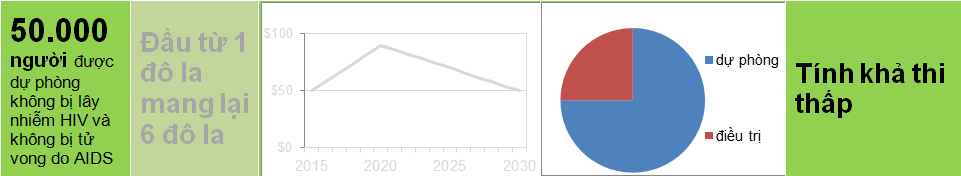


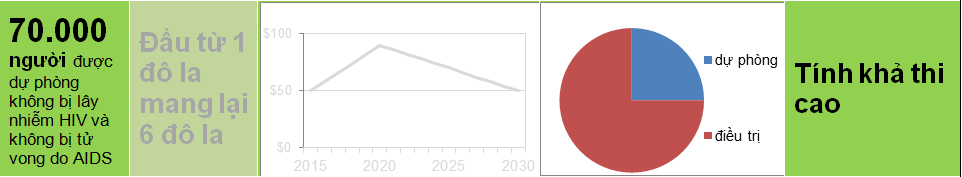


* 1. Đầu tư tăng nhanh trong 5 năm đầu, sau đó giảm dần qua các năm.


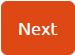


Khảo sát các tiêu chí lựa chọn chương trình phòng, chống HIV/AIDS tại Việt NamTop of Form

Hãy hình dung một tình huống khi bạn phải quyết định một chương trình bền vững để đối phó với HIV tại Việt Nam.

**Những tiêu chí nào sau đây bạn sẽ chọn?** Cả 2 giải pháp được đề xuất dưới đây đều có mức đầu tư trung bình 80 triệu đô la mỗi năm. Để dễ cho việc lựa chọn, chúng tôi đã để hình ảnh sáng hơn cho sự khác nhau giữa 2 giải pháp.


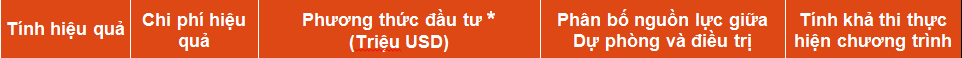


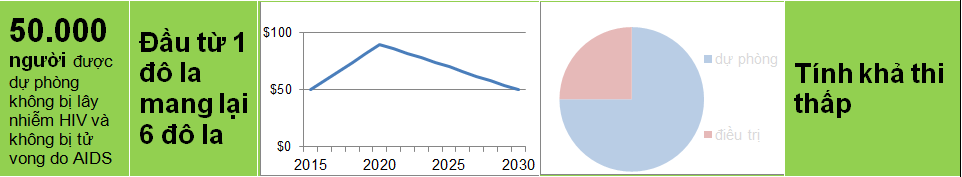


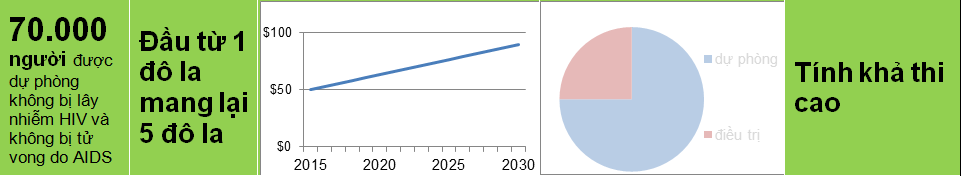


* 1. Đầu tư tăng nhanh trong 5 năm đầu, sau đó giảm dần qua các năm.

* 2. Đầu tư tăng dần qua các năm.


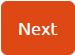


Khảo sát các tiêu chí lựa chọn chương trình phòng, chống HIV/AIDS tại Việt Nam

Tôi trân trọng cảm ơn anh/chị tham gia khảo sát này. Nếu anh chị có them bất kỳ câu hỏi nào, vui lòng liên hệ qua email: [a.safarnejad@student.maastrichtuniversity.nl](mailto:a.safarnejad@student.maastrichtuniversity.nl)
